# Preservation on calcium homeostasis is involved in mitochondrial protection of *Limonium sinense* against liver damage in mice

**DOI:** 10.4103/0973-1296.66935

**Published:** 2010

**Authors:** Xin-Hui Tang, Jin Chen, Xiao-Lan Yang, Li-Fang Yan, Jing Gao

**Affiliations:** 1 *Jiangsu Provincial Key Laboratory of Coastal Wetland Bioresources and Environmental Protection, Yancheng Teachers’ University, 50 Kaifang Road, Yancheng, 224002, China*; 2 *School of Pharmacy, Jiangsu University, 301 Xuefu Road, Zhenjiang, 212013, China*; 3 *School of Life Science, Nanjing University, 22 Hankou Road, Nanjing, 210009, China*; 4 *Burnett School of Biomedical Sciences, University of Central Florida, Orlando, FL 32816, USA*

**Keywords:** D-galactosamine, *Limonium sinense* (Girard) Ktze, lipopolysaccharide, mitochondrial Ca ^2+^ -ATPase activity, mitochondrial Ca ^2+^ overload

## Abstract

Mechanisms underlying the mitochondrial protection of *Limonium sinense* extracts (LSE) was studied in lipopolysaccharide and D-galactosamine (LPS/D-GalN) intoxicated mice. It was found that increased activities of serum aspartate aminotransferase and alanine aminotransferase induced by LPS/D-GalN were significantly inhibited by pretreatment with LSE. The obvious disruption of membrane potential, intramitochondrial Ca ^2+^ overload and suppression in mitochondrial Ca ^2+^ -ATPase activity induced by LPS/D-GalN were significantly blocked by pretreatment with LSE. It was concluded that mechanisms underlying protection of LSE against liver mitochondria damage might be related to the preservation on mitochondrial Ca ^2+^ homeostasis through the preservation on mitochondrial Ca ^2+^ -ATPase activity.

## INTRODUCTION

*Limonium sinense* (Girard) Ktze is a plant belonging to the Plumbaginaceae family and is mainly distributed along seashores and salts marshes in southern China, Ryukyus (Japan) and western Taiwan. Historically, both the roots and whole plants have been used as a folk medicine for the treatment of fever, hemorrhage, and menstrual disorders.[1] *L. sinense* extract (LSE) was reported to protect the hepatocytes against carbon tetrachloride (CCl_4_) and D-galactosamine (D-GalN) intoxication in rats.[2] Also, the major constituents found in the leaves and the roots of *L. sinense* were flavonoids.[3]

Evidence has accumulated that hepatocyte death is involved in liver injury and liver disease. Apoptosis and necrosis are crucial steps in the development of all kinds of liver injury, fibrosis, alcoholic liver disease and hepatitis.[4,5] It is recognized that mitochondria play a key role in controlling cell death and that mitochondria not only function as “power house” to provide ATP by oxidative phosphorylation but also play other roles such as the modulation of intracellular Ca^2+^ homeostasis, pH control and induction of apoptotic and excitotoxic cell death. Indeed, mitochondrial dysfunction contributes to a great number of human and animal diseases.[6]

Recently, our study confirmed the hepatoprotective effects of LSE in both CCl_4_ and lipopolysaccharide (LPS)/D-GalN intoxicated mice and found that LSE could block the decrease in the mitochondrial membrane potential and sensitivity to mitochondrial swelling and regulate the expression of voltage-dependent anion channels (VDAC), an important channel protein on the outer membrane of mitochondria in both CCl_4_ and LPS/d-GalN-intoxicated mice, which demonstrated that the mechanism underlying the hepatoprotection of LSE might be related to the protection of liver mitochondria though stabilizing the expression of mitochondrial VDAC.[7,8]

It is generally accepted that the concentration of cytosolic free Ca^2+^ plays an important role in the regulation of many hepatocyte functions.[9] It has been found that damage to hepatocytes is always associated with an increased influx of Ca^2+^ down the steep electrochemical gradient that exists between the inside and the outside of the cells.[10,11] Also, various hepatotoxicated substances can result in hepatocellular Ca^2+^ overload, which can activate the mitochondrial Ca^2+^ uniporter in the mitochondrial inner membrane and induce a mitochondrial Ca^2+^ influx.[12] Excessive intramitochondrial Ca^2+^ leads to the opening of mitochondrial permeability transition pore (PTP), a channel at the contact sites between the inner and outer mitochondrial membranes, which allows solutes of molecular weights greater than 1.5 kDa to pass between the mitochondrial matrix and the cytoplasm and causes equilibration of ions within the matrix and the cytosol, dissipating the membrane potential, uncoupling the respiratory chain. The volume disregulation following the opening of the PTP results in the swelling of the matrix, leading to outer membrane disruption and the release of proapoptotic proteins such as cytochrome c and apoptosis-inducing factor (AIF) into the cytosol, ultimately contributing to cell death.[12,13] Liver mitochondrial Ca^2+^ overload occurred in hepatotoxicated mice and drugs could protect the mitochondria though maintaining the mitochondrial Ca^2+^ content.[14–17] However, the effect of LSE on liver mitochondria Ca^2+^ homeostasis is unknown. In the present study, we address the possible mechanisms about mitochondrial Ca^2+^ handling involved in the mitochondrial protection of LSE in LPS/d-GalN-intoxicated mice.

## MATERIALS AND METHODS

### Plant material

Roots of *L. sinense* were collected at the Yancheng seabeach in China and identified by Mr. Yao Gan (Institute of Botany of Jiangsu Province, Chinese Academy of Sciences) in December 2005. A voucher specimen (No. 051205) was deposited in Jiangsu Provincial Key Laboratory of Coastal Wetland Bioresources and Environmental Protection, Yancheng Teachers’ University (Yancheng, China).

LSE was prepared as follows. Dried cut roots of *L. sinense* (100 g) were extracted with water (800 ml) by reflux for 2 h three times, and the extracts combined and subjected to evaporation to obtain 32.89 g (yield: 32.89% w/w) of crude LSE.

### Chemicals

D-galactosamine (D-GalN), lipopolysaccharide (LPS), Fura-2/AM, rhodamine 123 (Rh123), succinate, rotenone were purchased from Sigma (St. Louis, MO, USA). Aspartate aminotransferase (AST), alanine aminotransferase (ALT) and ATPase test kits were purchased from Nanjing Jiancheng Bioengineering Institute (Nanjing, China). All other chemicals were of high purity, obtained from commercial sources.

### Animals

Male ICR mice (Experiment Animal Center of Nanjing Medical University, Nanjing, China, certificate No. SCXK 2002-0031) weighing 20 ± 2 g were used. The mice were housed at a temperature of 20–25°C under a 12-h light/dark cycle with 50% of relative humidity and kept in filtered, pathogen-free air. They were fed on commercial laboratory chow and given tap water. This study complied with current ethical regulations on animal research in Jiangsu University and Yancheng Teacher’s University and all the mice used in the experiments were treated humanely. Procedures were performed according to the recommendations of the institutional animal care committee of Jiangsu University and Yancheng Teacher’s University.

### Lipopolysaccharide and D-galactosamine induced hepatotoxicity in mice

LSE (100, 200 and 400 mg/kg) was administrated intragastrically to three groups of eight mice once daily for five consecutive days followed by a final treatment of LPS/d-GalN (10 μg/kg, 600 mg/kg, ip, respectively). Two other groups were treated as follows: a group of non-intoxicated animals (normal group) received vehicles (10 ml/kg, ip) only, and a model group (injury group) received saline (20 ml/kg, ig) for 5 days followed by LPS/d-GalN treatment. Twelve hours after the final treatment, blood was collected and mice were euthanized. The serum was obtained by centrifugation at 3000 *g* for 20 min at room temperature. After blood draining, liver sections were taken and fixed in 4% neutral-buffered formalin and prepared for examination under a photomicroscope. Mitochondria were removed and separated from the livers to evaluate their function. The remaining livers were homogenized to analyze liver lipid peroxidation levels.

### Aminotransferase activity determination

Serum ALT and AST activities, markers for hepatotoxicity, were determined using an automatic analyzer (Hitachi 7600, Hitachi High Technologies Corp., Tokyo, Japan).

### Lipid peroxidation level determination

Liver lipid peroxidation level was analyzed by measuring malondialdehyde formation using the thiobarbituric acid method.[18]

### Isolation of liver mitochondria

Mitochondria were prepared from mouse livers according to the method of Apprille.[19] In brief, mouse livers were excised, homogenized in isolation buffer containing 225 mmol/l d-mannitol, 75 mmol/l sucrose, 0.05 mmol/l ethylene diamine tetraacetic acid (EDTA) and 10 mmol/l hydroxymethyl aminomethane hydrochloride (Tris-HCl) (pH 7.4) at 4°C. The homogenates were centrifuged at 600 *g* for 5 min and supernatants were centrifuged at 8800 *g* for 10 min at 4°C. The pellet was washed twice with the same buffer. Protein concentration was determined using Coomassie Brilliant Blue.[20]

### Measurement of mitochondrial membrane potential

Mitochondrial membrane potential (MMP) was evaluated according to the method of Emaus,[21] from the uptake of the fluorescent dye Rh123, which accumulates electrophoretically into energized mitochondria in response to their negative inside membrane potential. Liver mitochondria (0.5 mg protein/ml), isolated from mouse livers of the various groups, were prepared in the assay buffer containing 225 mmol/l of mannitol, 70 mmol/l of sucrose and 5 mmol/l of HEPES (*N*-2-hydroxyethylpiperazine-*N*’-2-ethanesulfonic acid), pH 7.2. MMP was assessed spectrophotometrically with excitation at 505 nm and recording at 534 nm by Hitachi 850 fluorescence spectrophotometer after the addition of 0.3 μmol/l of Rh123 at 25°C. Membrane potential was calculated by the relationship: MMP = –59 log [Rh123]in/[Rh123]out, assuming that the distribution of Rh123 between mitochondria and the medium follows the Nernst equation.[22]

### Measurement of mitochondrial Ca^2+^

The intramitochondrial Ca^2+^ level was assayed by the change in fluorescent intensity (F) of the Ca^2+^ indicator dye fura-2. To load mitochondria with the fluorescent Ca^2+^ indicator fura-2, 0.5 mg protein/ml of mitochondria isolated from various groups’ mice livers was incubated for 30 min at 30°C in a suspension medium containing 125 mmol/l of sucrose, 65 mmol/l of KCl, 5 mmol/l of HEPES and 1 μmol/l of fura-2/AM, pH 7.4, and then washed twice with the medium without the dye to eliminate free fura-2/AM. The final mitochondrial pellet was diluted in the suspension medium to obtain a protein concentration of 0.5 mg/ml. For every sample, the *F* of fura-2-loaded mitochondria was recorded on a Hitachi 850 fluorescence spectrometer at an excitation wavelength of 340 nm and an emission wavelength of 510 nm. *F*_max_ was determined by adding 0.4% Triton X-100 and 1 mmol/l of CaCl _2_ to the mitochondrial suspension; *F*_min_ was measured by adding 10 mmol/l of ethylene glycolbis(2-aminoethyl ether)-*n,n,n′,n′* tetraacetic acid (EGTA) to the above system. The intramitochondrial Ca^2+^ content was calculated as follows:*K*_d_ (*F* – *F*_min_)/(*F*_max_ – *F*).[23]

### Analysis of mitochondrial Ca^2+^ -ATPase activity

Liver mitochondria (0.5 mg protein/ml) were prepared in the assay buffer containing 50 mmol/l of Tris-HCl, 75 mmol/l of KCl, 0.4 mmol/l of EDTA and 6.0 mmol/l of MgCl _2_, pH 7.4. Ca^2+^ -ATPase activity was assayed by measuring phosphate release according to the protocol in the ATPase kit (Jiancheng Bioengineering Institute). One unit of the specific activity of the ATPase was defined as one micromole of inorganic phosphorus released from 1 mg of protein within 1 h (μmol Pi/mg protein/h).

### Statistical analysis

Differences among all the groups were analyzed by one-way analysis of variance, followed by SNK-*q*-test using SPSS 10 software.

## RESULTS

### Effect of *L. sinense* extract on serum alanine aminotransferase and aspartate aminotransferase activities and liver lipid peroxidation level

As shown in Table 1, LPS/d-GalN treatment induced a remarkable elevation in both serum ALT and AST activities when compared with the normal level. Also, the liver lipid peroxidation level in LPS/d-GalN-intoxicated mice was obviously increased. However, treatment with 100–400 mg/kg LSE significantly blocked the above changes, especially the 200 and 400 mg/kg LSE treatment, which maintained the enzyme activities and liver lipid peroxidation level almost at normal level.

**Table 1:** Effect of LSE on LPS/D-GalN-induced elevation in serum aminotransferases and liver lipid peroxidation levels in mice

Groups	ALT activity (U/l)	AST activity (U/l)	Lipid peroxidation (nmol malondialdehyde/mg/h)
Normal	24.14 ± 4.74	97.29 ± 18.2	3.80 ± 0.45
LPS/d-GalN	3232.43 ± 886.47**	3019.5 ± 1354.47**	11.62 ± 0.98**
100 mg/kg LSE	1182.83 ± 681.7**##	1098.83 ± 558.8**##	8.72 ± 0.89**#
200 mg/kg LSE	208.29 ± 109.39##	227.5 ± 130.94##	4.93 ± 0.78##
400 mg/kg LSE	90.5 ± 39.89##	131.43 ± 20.81##	4.16 ± 0.42##

Mice were divided into five groups: normal, LPS/d-GalN, 100, 200, 400 mg/kg LSE groups. Blood was collected and livers were taken from LPS/d-GalN and different LSE groups, 12 h after the intraperitoneal injection with LPS (10 μg/kg) and d-GalN (600 mg/kg). Serum ALT and AST activities and liver lipid peroxidation level were determined. Each value represents mean ± SD of eight mice.

** *P* < 0.01 compared with normal group

# *P* < 0.05

## *P* < 0.01, compared with LPS/d-GalN group

### Histological observation

**Figure 1:**
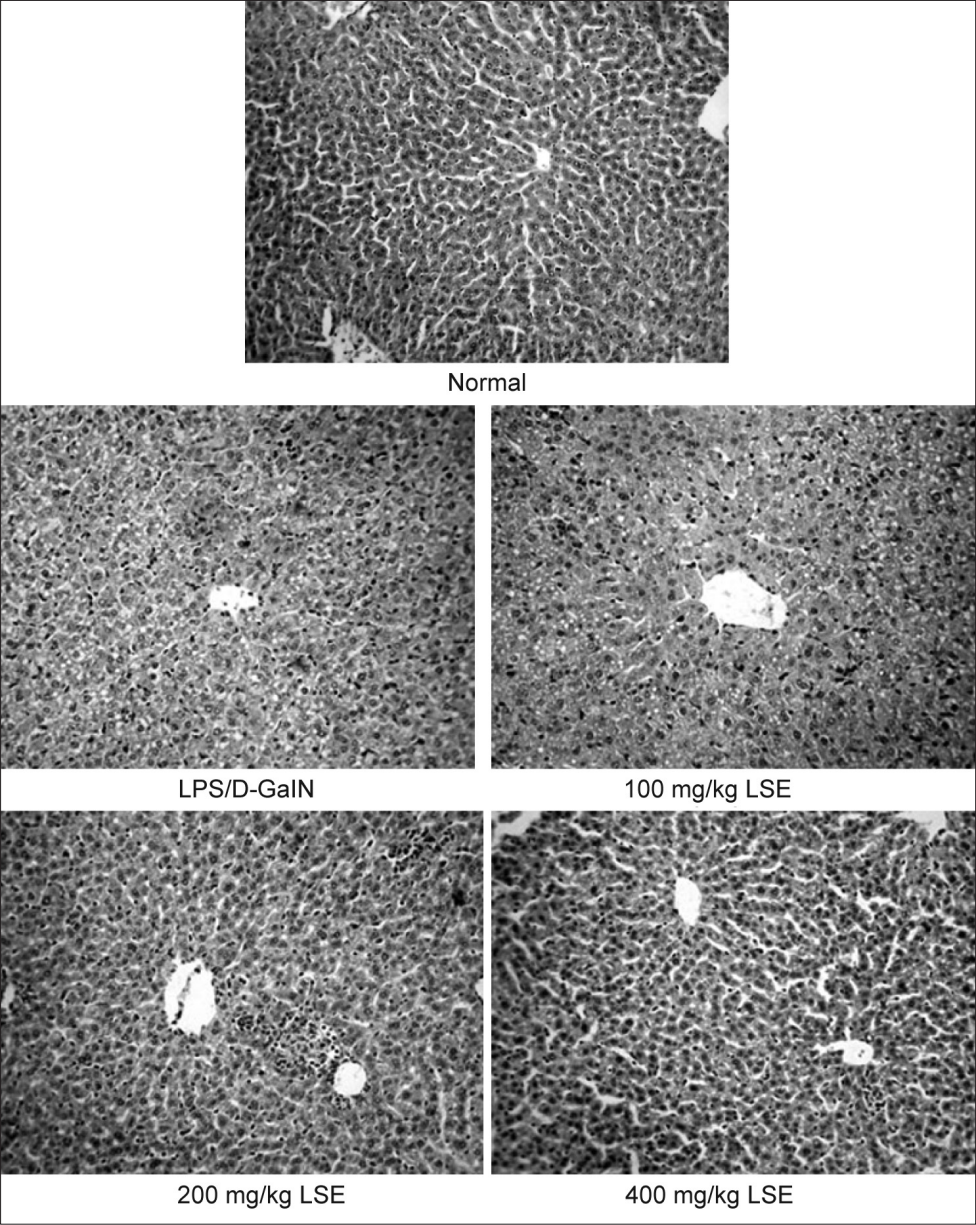
Microphotographs of liver of mice (H and E). Mice were divided into five groups: normal, LPS/D-GalN, 100, 200, 400 mg/kg LSE groups. Livers from LPS/D-GalN and different LSE groups were taken 12 h after the intraperitoneal injection with LPS (10 μg/kg) and D-GalN (600 mg/kg) and regularly prepared for the examination under microscope (×100)

Compared with the normal group, obvious structure changes such as massive fatty change, gross necrosis, broad infiltration of the lymphocytes and kupffer cells around the central vein and loss of cellular boundary were observed in LPS/d-GalN-insulted mice. However, the histological pattern of the livers of the mice treated with LSE showed only mild degrees of fatty change, necrosis and lymphocyte infiltration [Figure 1].

### Effect of *L. sinense* extract on mitochondrial membrane potential dissipation

**Figure 2:**
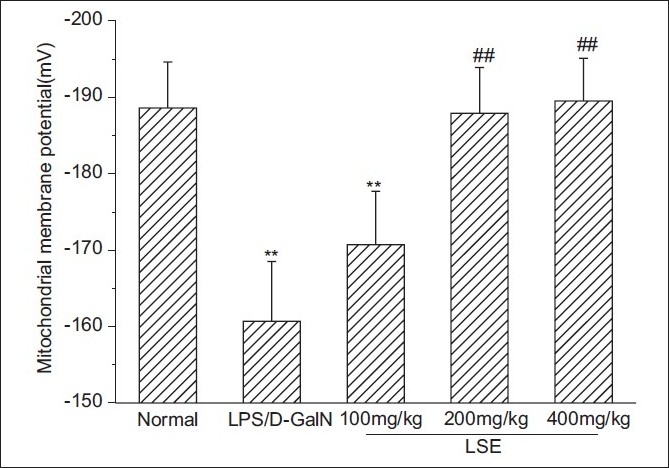
Prevention of LSE on MMP dissipation induced by LPS/D-GalN. Livers from LPS/D-GalN and different LSE groups were taken 12 h after the intraperitoneal injection with LPS (10 ìg/kg) and D-GalN (600 mg/kg). Liver mitochondria were isolated and MMP was determined using Rh123. Each value represents mean ± SD of eight mice. ^**^P < 0.01 compared with normal group. ^##^*P* < 0.01 compared with LPS/D-GalN group

Under the present experimental condition, the MMP of normal mice was –188.6 ± 5.9 mV, which dropped to –160.7 ± 7.8 mV (*P* < 0.01) when mice were intraperitoneally injected with LPS/d-GalN [Figure 2]. At a dose of 200 or 400 mg/kg of LSE, the MMP was restored to that observed for normal mice. While at a dose of 100 mg/kg, the MMP increased compared with that of LPS/d-GalN group, but this increase was not statistically significant.

### Effect of *L. sinense* extract on mitochondrial Ca^2+^ overload

**Figure 3:**
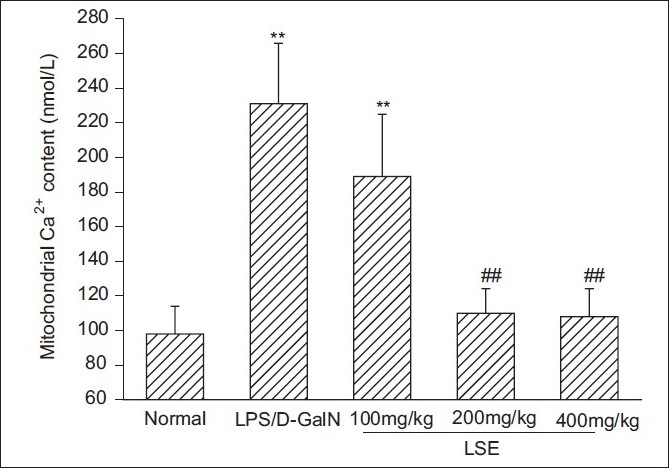
Effect of LSE on liver mitochondrial calcium content in mice treated with LPS and D-GalN. Livers from LPS/D-GalN and different LSE groups were taken 12 h after the intraperitoneal injection with LPS (10 ìg/kg) and D-GalN (600 mg/kg). Liver mitochondria were then isolated and mitochondrial free calcium content was determined using fura-2. Values represent mean ± SD of eight mice.^**^P < 0.01 compared with normal group. ^##^*P* < 0.01 compared with LPS/D-GalN group

Measurement of Ca^2+^ content using the fluorescent probe fura-2 showed that intramitochondrial Ca^2+^ content in LPS/d-GalN-intoxicated mice was much higher (2.4-fold) than that in normal mice. However, the rise in Ca^2+^ handling level induced by LPS/D-GalN was effectively inhibited by pretreatment with various concentration of LSE, and the inhibitory rates in the 100, 200 and 400 mg/kg LSE groups reached 31.6, 89.9 and 90.5%, respectively Figure 3.

### Effect of *L. sinense* extract on the mitochondrial Ca^2+^ -ATPase activity

**Figure 4:**
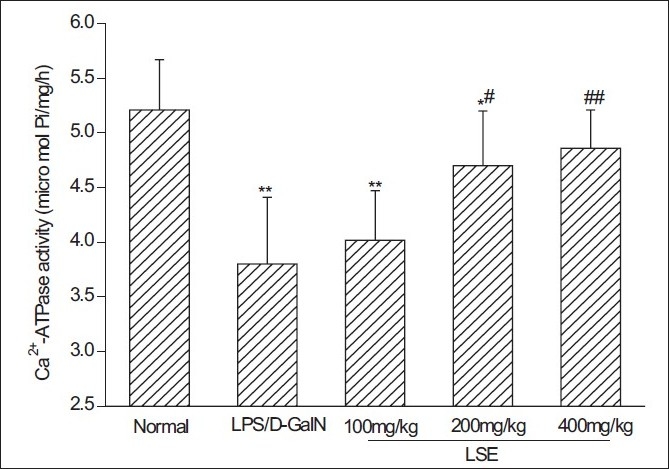
Effect of LSE on liver mitochondrial Ca2+-ATPase activity in mice treated with LPS and D-GalN. Livers from LPS/D-GalN and different LSE groups were taken 12 h after the intraperitoneal injection with LPS (10 ìg/kg) and D-GalN (600 mg/kg). Liver mitochondria were then isolated and mitochondrial Ca^2+^-ATPase activity was determined according the protocol in the kit. Values represent mean ± SD of eight mice.^*^P < 0.05, ^**^P < 0.01 compared with normal group. ^#^*P* < 0.05, ^##^*P* < 0.01 compared with LPS/D-GalN group

The effect of LSE on mitochondrial Ca^2+^ -ATPase activity is shown in Figure 4. Mitochondrial Ca^2+^ -ATPase activity in LPS/D-GalN-intoxicated mice (3.8 ± 0.6 μmol Pi/mg protein/h) was obviously lower than that in normal mice (5.2 ± 0.5 μmol Pi/mg protein/h). However, the LSE of various concentrations obviously blocked the defect in mitochondrial Ca^2+^ -ATPase activity. The inhibitory rates of 100, 200 and 400 mg/kg of LSE reached 14.3, 64.3 and 78.6%, respectively.

## DISCUSSION

*L. sinense* (Girard) Ktze is a folk medicine popularly used as a remedy for bleeding, piles, fever, hepatitis, diarrhea, bronchitis and other disorders.[24] As reported by Lin, the major constituents found in the leaves and the roots of *L. sinense* are flavonoids.[3] Our previous study showed that LSE could prevent both CCl_4_ and LPS/d-GalN-induced liver damage and that the hepatoprotection is related to its protection of liver mitochondria though stabilizing the expression of mitochondrial VDAC.[7,8] However, the mechanism about mitochondrial Ca^2+^ handling involved in the mitochondrial protection of LSE is still not known.

Liver injury induced by LPS/D-GalN is a well-characterized system of acute hepatic failure and usually used for screening of anti-hepatotoxic and/or hepatoprotective activity of drugs.[25,26] In this study, LPS/D-GalN-induced liver injury was used to study the mechanisms underlying the mitochondrial protection of LSE. It was found that increases in the activities of serum AST and ALT induced by LPS/D-GalN were significantly inhibited by oral pretreatment with 100, 200 or 400 mg/kg LSE. Morphological observation further confirmed the hepatoprotective effects of LSE. Meanwhile, the obvious disruption of membrane potential in LPS/D-GalN-intoxicated mice was siginificantly blocked by pretreatment with LSE, which coincided with our previous results[8] and demonstrated that LSE has protective function on liver mitochondria against damage caused due to LPS/D-GalN.

Previous reports have suggested that there are several steps in the mechanism by which GalN induces hepatocytes’ death, and loss of Ca^2+^ homeostasis in damaged hepatocytes is one of the most important steps. Moreover, the hepatocellular Ca^2+^ overload can activate the mitochondrial Ca^2+^ uniporter in the mitochondrial inner membrane and eventually cause swelling of the mitochondrial matrix, dissipation of mitochondrial membrane potential, release of Ca^2+^ and proapoptotic factors, and finally induce cell death, which has been considered as one of the important mechanisms in liver injury.[4]

Ca^2+^ homeostasis was evaluated by measuring intramitochondrial Ca^2+^ content in LPS/D-GalN-intoxicated mice with or without pretreatment with LSE. The present results show that 200 and 400mg/kg LSE effectively suppressed the intramitochondrial Ca^2+^ overload induced by LPS/D-GalN, which suggests that LSE could protect liver mitochondria against the toxicity of LPS/d-GalN by preserving the mitochondrial Ca^2+^ homeostasis. We can speculate that the suppression of LSE on LPS/D-GalN-induced intramitochondrial Ca^2+^ overload might result in a blockage of mitochondrial calcium influx, which in turn inhibits the swelling of the mitochondrial matrix, dissipation of mitochondrial membrane potential and release of proapoptotic factors through prohibiting PTP opening, and finally blocking the hepatocyte death.

It was also believed that cells maintain a cytosolic Ca^2+^ homeostasis through the action of Ca^2+^ -ATPase located on the plasma membrane. This enzyme uses the energy of ATP to extrude cytoplasmic Ca^2+^ against a large concentration gradient into the extracellular space. Also, more and more evidences suggest that mitochondria are particularly important in controlling cytoplasmic Ca^2+^ levels under pathological conditions. Ca^2+^ -ATPase located on the mitochondrial membrane can take up and retain large quantities of Ca^2+^ to buffer cytosolic Ca^2+^ levels and prevent damage to a cell.[16] Our present studies show that LSE obviously blocked the decrease in mitochondrial Ca^2+^ -ATPase activity induced by LPS/D-GalN. We therefore speculate that the maintenance of the mitochondrial Ca^2+^ homeostasis by LSE may be related to its preservation of mitochondrial Ca^2+^ -ATPase activity.

## CONCLUSION

In summary, the results in the present study demonstrate that mechanisms underlying protective function of LSE on liver mitochondria against damage by LPS/D-GalN in mice could be related to the preservation on mitochondrial Ca^2+^ homeostasis through the preservation on mitochondrial Ca^2+^ -ATPase activity, which reveals a new mechanism of the mitochondrial protective effect of LSE.
